# Co-Designing a Digital Solution for Decreasing Loneliness and Social Isolation Among Older People in Sweden: Explorative Study

**DOI:** 10.2196/78213

**Published:** 2025-11-21

**Authors:** Caroline Eklund, Viktoria Zander, Annelie K Gusdal, Charlotta Åkerlind, Petra von Heideken Wågert

**Affiliations:** 1 School of Health, Care and Social Welfare Mälardalen University Västerås Sweden; 2 School of Health, Care and Social Welfare Mälardalen University Eskilstuna Sweden

**Keywords:** action research, digitalization, eHealth, healthy aging, internet intervention, participatory research design

## Abstract

**Background:**

Older people are particularly vulnerable to loneliness and social isolation due to common age-related changes. The ability to maintain social relationships is considered important for health and well-being and is an essential aspect of healthy aging. The use of information and communication technology has been shown to promote social connectedness and social support among older people; however, many existing solutions require already established contacts and are not developed based on expressed needs among older people experiencing loneliness or social isolation.

**Objective:**

The overarching aim of this study was to develop a prototype of a health and welfare technology intervention for decreasing loneliness and social isolation among older people.

**Methods:**

This study describes an iterative participatory design process conducted in 3 phases to create a prototype of a digital intervention for decreasing loneliness and social isolation in older people through participatory design procedures with several key stakeholders. Phase I explored social service staff’s perceptions of how social isolation and experiences of loneliness among older people can be reduced by health and welfare technology. Data were collected by conducting 2 workshops (WSs) with social service staff. Phase II explored needs related to loneliness and social isolation perceived by older people and how these needs may be targeted by using health and welfare technology. Data were collected through 2 semistructured interviews and 2 WSs. In phase III, 3 co-design WSs brought together older people with experience of loneliness or social isolation, social service staff, and IT experts to collaboratively generate and refine design ideas.

**Results:**

The main result of the study was the development of a paper prototype of the Fik@ room, which is a digital solution supporting social interaction among older people experiencing loneliness or social isolation. Key needs identified include the desire for low-threshold opportunities to connect, flexible modes of communication, and a safe and welcoming environment. These needs directly informed the final design. The main feature of the prototype is to facilitate social interaction in a digital break room with coffee tables where users can interact via video, audio, or chat. Other features are a bulletin board for announcements about interactions inside or outside of the Fik@ room, as well as a profile page.

**Conclusions:**

The iterative co-design process ensured that the solution was grounded in user needs, which may have contributed to the usability and acceptability of the Fik@ room and potentially effective in reducing loneliness and social isolation among older people. Future research should focus on evaluating effects in a real-world setting.

## Introduction

The rapidly growing aging population poses a global demographic challenge [1,2]. This challenge is strongly tied to the health and well-being of the older population, and these qualities are influenced by multiple factors, including psychosocial factors such as social isolation and loneliness [3-5]. Loneliness and social isolation are multidimensional concepts that are sometimes used interchangeably, yet they have distinct meanings. Although there is no single universally agreed-upon definition of loneliness, one of the most widely cited definitions is provided by Perlman and Peplau [6], who conceptualize loneliness as “the unpleasant experience that occurs when a person’s network of social relations is significantly deficient in quantitatively or qualitatively”. This definition highlights that loneliness is inherently subjective and negative, which clearly differentiates it from objective social isolation, which may also be voluntary. While loneliness relates to a subjective feeling of lost friendship and support as a result of few or insufficient social relationships and interactions [7], social isolation is usually characterized as a lack of social networking and social support [8]. Although social isolation may be voluntary, loneliness is always involuntary by definition. Regardless of which term is used, loneliness in the older population elicits concern, and loneliness has been described as being more prominent among people aged 75-85 years than among people aged 57-65 years [9]. The estimated prevalence of loneliness in older adults (aged 60 years and older) has varied from 7% [10] to as much as 49% in the oldest adults (aged 80 years and older) [11].

The ability to maintain social relationships is considered important for health and well-being and is an essential part of healthy aging [1,2]. Few or insufficient social relationships are threats to the mental and physical health of the older population and are associated with lower levels of self-rated physical health [9], as well as with a range of health outcomes, such as malnutrition, hypertension, disrupted sleep, frailty, and increased mortality [12-16]. The biological mechanisms that have been proposed to mediate the link between loneliness and poor health conditions include genetic, neural, and hormonal mechanisms related to various physiological functions, including dysregulated hypothalamic‒pituitary‒adrenal axis function and immune function [13,17]. Moreover, experienced loneliness has been shown to predict nursing home admissions [18] and is related to poor emotional and cognitive health, ranging from psychoses, personality disorders, depression, and suicide to impaired cognitive performance and the risk of Alzheimer disease [19].

Older people are particularly vulnerable to loneliness and social isolation due to common age-related changes and losses, such as poor health status, poor functional status, poor vision, and loss of hearing [20]. Moreover, older people use the internet to a lesser degree than younger people do, as they tend to prefer face-to-face and telephone contact [21]. However, this is only one of several factors influencing lower levels of social technology use. Barriers also include low perceived self-efficacy, fear of technology, lack of social capital, and physical limitations, which may hinder older peoples’ motivation and ability to engage with social technology [22]. This indicates that those individuals not able to be active outside of their homes have risks of feeling excluded in two manners, including both by physical contact and by digital exclusion from a digitally dominated society [23]. The use of information and communication technology (ICT) has been shown to promote social connectedness and social support among older people through the development and maintenance of social networks. ICT use has been shown to decrease feelings of loneliness [24] and social isolation and foster well-being, life satisfaction, and self-worth, as well as enhance social support, social contacts, and social networks [3,25], with a particular favoring of videoconferencing [26]. However, it is important that the development of ICT considers the specific needs of older people [22,25,27]. Models such as the Unified Theory of Acceptance and Use of Technology [28] and the Senior Technology Acceptance Model [29] highlight that perceived usefulness and ease of use are important for adoption of new technologies among older people, but that social influence, prior experience, trust, and facilitating conditions (eg, self-efficacy for use, attitudes, age-specific considerations like physical and cognitive abilities, support, and accessibility) also play crucial roles in determining whether older people will engage with new technology. These models are particularly relevant when designing interventions aimed at reducing loneliness and social isolation, as successful adoption and implementation rely not only on addressing functional needs but also on aligning with users’ expectations, capabilities, and social contexts.

One possible method of achieving success when developing interventions for a specific group is to involve potential end users during the development process [30]. Participatory approaches, such as co-design by involvement of end users and stakeholders collaborating in the solution development process, are common in health care and have been applied in the development of a number of ICT tools to support self-care for people with chronic conditions [31,32]. Co-design in research has been described as “doing research ‘with’ or ‘by’ the public rather than ‘to’, ‘about’, or ‘for’ the public” [33] and has been argued to have the potential to improve the quality, relevance, and impact of health research [30,33]. Furthermore, there are also moralistic arguments for participatory research and co-design, as the public has the right to be involved in any publicly funded research that may impact them [33]. Although co-design principles have been used less with older people, there is currently a growing interest in involving older people in the development of solutions to maintain well-being and independence, as well as to promote healthy aging [32]. ICT can support safe and active aging; however, its acceptance and use can be suboptimal without consideration of the end users’ needs and preferences [34]. Research suggests that ICT solutions that have been developed in collaboration with end users can have a positive impact on a number of health outcomes, such as the ability to cope with a disease, disease control, reductions in falls, improvements in the care experience, better access to health care, improved patient satisfaction, and reductions in costs [32]. Moreover, the solutions have been perceived to be more applicable and acceptable to end users [30], which may facilitate implementation.

A previous study identified loneliness and social isolation as being important contributing factors to decreased health and well-being among older persons; furthermore, health and welfare technology developed for older people was identified as having the potential to reduce loneliness [35]. Health and welfare technology comprises the umbrella term ICT and is defined as “a technology-based intervention that aims at maintaining or promoting health, well-being, quality of life, and increasing efficiency in the service delivery system of welfare, social and health care services, while improving working conditions of the staff” [36]. The development of a health and welfare technology solution for decreasing loneliness and social isolation via co-design may increase the acceptability of this solution to potential end users. Moreover, by involving the staff who meet these people in the design, the solution may be easier to implement in the organization. Experts within the field, such as researchers and system developers, may also contribute to broadening the perspective to suggest solutions and to ensure an evidence base.

The overarching aim of this study was to develop a prototype of a health and welfare technology intervention for decreasing loneliness and social isolation among older people. The specific aims of the different phases of the study were (1) to explore social service staffs’ perceptions of how experiences of loneliness and social isolation among older people can be reduced by health and welfare technology; (2) to explore needs related to loneliness and social isolation perceived by older people and how these needs may be targeted by using health and welfare technology; and (3) to, in co-design with older people, social service staff, system developers and researchers, develop a prototype of a health and welfare technology intervention for social interaction to reduce experienced loneliness and social isolation among older people.

## Methods

### Overview

This study had an explorative design using a participatory research design based on a model for experience-based co-design by Robert [37]. According to the 3 specific aims, the study was conducted in 3 phases with separate data collection methods and analyses of the data and results. We used the COREQ (Consolidated Criteria for Reporting Qualitative Research) item checklist for reporting the study (Multimedia Appendix 1 [38]).

Robert [37] described the process of experience-based co-design in six stages: (1) setting up the design, (2) engaging staff and gathering experiences, (3) engaging patients/staff and collecting experiences, (4) gathering patients and staff to share experiences and begin the co-design process, (5) providing detailed co-design activities, and (6) gathering together again for celebration, review and renewal. Stage 1 included establishing governance and project management arrangements and was conducted prior to data collection by a core team consisting of researchers, social service staff, a system developer, and the intended workshop (WS) leader. Stage 6 was not included in this study.

The data were mainly collected through WSs with key stakeholders, including social service staff, older persons, system developers, and researchers. Interviews in phase II were conducted by the second author, the WSs in phase I were conducted by authors 3 and 4, and the WSs in phases II and III were led by an experienced WS leader from an IT company. All researchers/authors have previous experience in qualitative methodology and conducting interviews. Participants received no compensation for participating in any of the phases.

In phase I, the WSs were conducted at the researchers’ university and lasted approximately 3 hours each. In phase II, the WSs were held in a conference room at the municipality’s Family Caregiver Centre, which is separate from the university, with a duration of approximately 2 hours per WS. The individual interviews in phase II were conducted at the researchers’ university and lasted for approximately one hour each. The phase III WSs were again conducted at the researchers’ university, each lasting about 2 hours.

**Figure 1:**
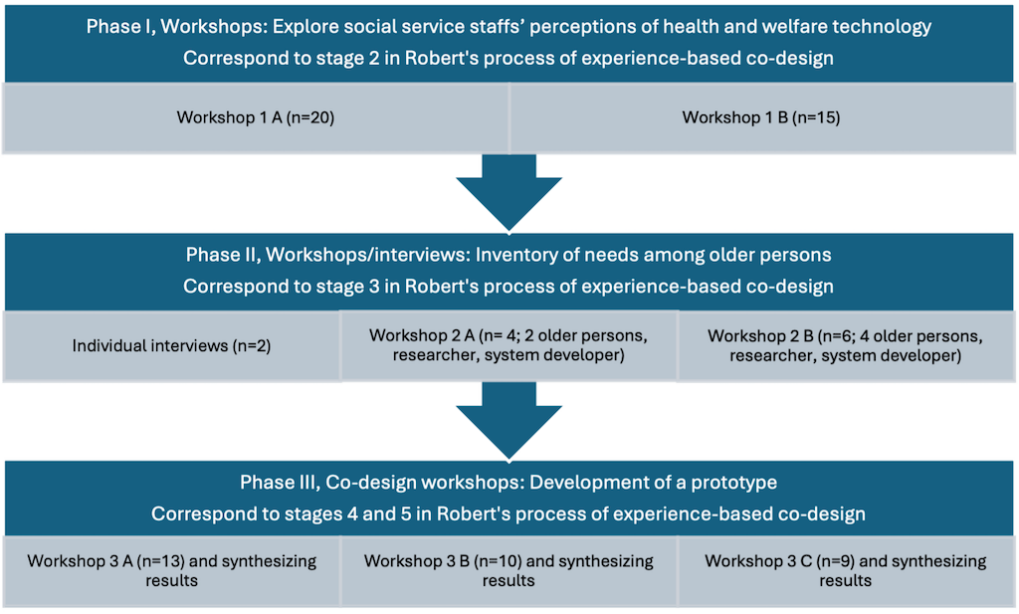
The 3 phases for data collection, the number of participants in each workshop, and how the phases are related to Robert’s process of experience-based co-design.

The process was iterative, and the results were synthesized between WSs and phases. Figure 1 shows an overview of the WSs included in each phase. Methods for each phase are presented below.

### Phase I

The aim of phase I was to explore social service staff’s perceptions of how experiences of loneliness and social isolation among older people can be reduced by health and welfare technology. An invitation to participate in 2 WSs was sent to social service organizations in 2 municipalities in the Mideast of Sweden. The invitation was directed to staff in the home care service, care managers, and care unit managers to discuss current research and brainstorm what health and welfare technology is suitable and feasible for use in the municipal care setting. In the invitation, it was expressed that it was desirable that the participants be present on both occasions. Interested people contacted the researchers. Twelve people participated in both WSs 1 A and 1 B. Figure 1 provides the total number of participants. Table 1 shows the distribution of participants according to municipality and profession. The 2 WSs were conducted during the autumn of 2018 by authors 3 and 4.

In WS 1 A, a presentation introduced the participants to the body of research within digital interventions for decreasing loneliness and social isolation among older people through health and welfare technology. The researchers who were leading the WS presented an overview of the published research in the field, including interventions organized into 4 categories: videoconference/telephone, education (eg, in the use of computers and social platforms, eg, Facebook), robots for social interaction, and TV games. Afterwards, the participants were divided into 4 groups that discussed how these digital interventions could be implemented in their organizations and the potential content and use of digital interventions for decreasing experienced loneliness and social isolation among older people. The groups were created to include persons of different sexes, professions, and years of employment. Notes were taken regarding key messages presented at the end of the WS by one of the researchers.

WS 1 B started with a summary of WS 1 A, in which the participants concluded that the intervention of interest was a web-based platform for socializing, with the opportunity for physical meetings, after which the WS continued with discussions in 4 groups. The discussion departed from 4 questions: “What should a web-based platform for decreasing experienced loneliness contain?” “How should the intervention be implemented?” “Which layout and name could be appropriate for the web-based platform?” and “How could the target population be reached?” Additionally, there was time for general discussions on the topic.

The WSs were not audio recorded because some participants opposed recording, as they felt it would inhibit their participation. Data were collated through field notes. The collected data were analyzed via categorization of the notes that were taken during the WSs.

**Table 1:** Characteristics of participants in the workshops and individual interviews conducted in phases I-III of the study. Participants were social service staff working with older people and contributed with their perceptions of how health and welfare technology could help reduce loneliness and social isolation among older people, older people (68-91 years of age with experience of loneliness or social isolation), system developer, researchers, and an IT pedagogue.

Profession			Municipality X/Y, n/n		Participants, n
**Phase I: Workshop 1 A (n=20, female/male: 16/4)**					
	Care manager/care unit manager	4/6		10	
	Occupational therapist	0/1		1	
	Home care staff	3/5		8	
	Technical coach	1/0		1	
**Phase I: Workshop 1 B (n=15, female/male: 11/4)**					
	Care unit manager	2/4		6	
	Home care staff	3/3		6	
	Technical coach	1/0		1	
	Strategist	0/2		2	
**Phase II: Individual interviews (female/male: 1/1)**					
	Older people	N/A^a^		2	
**Phase II: Workshop 2 A (female/male: 1/1)**					
	Older people	N/A		2	
**Phase II: Workshop 2 B (female/male: 1/1)**					
	Older people	N/A		4	
**Phase III: Workshop 3 A (n=13, female/male: 12/1)**					
	Older people	N/A		4	
	Social service staff	N/A		4	
	IT pedagogue	N/A		1	
	Researchers	N/A		2	
	Research administrator	N/A		1	
	System developer	N/A		1	
**Phase III: Workshop 3 B (n=10, female/male: 10/0)**					
	Older people	N/A		2	
	Social service staff	N/A		3	
	IT pedagogue	N/A		1	
	Researchers	N/A		2	
	Research administrator	N/A		1	
	System developer	N/A		1	
**Phase III: Workshop 3 C (n=9, female/male: 9/0)**					
	Older people	N/A		2	
	Social service staff	N/A		3	
	Researchers	N/A		2	
	Research administrator	N/A		1	
	System developer	N/A		1	

^a^Not applicable.

### Phase II

The purpose of phase II was to explore needs related to loneliness and social isolation perceived by older people and how these needs may be targeted by using health and welfare technology. The data were collected during the spring of 2019.

For this phase, 6 older people with experiences of loneliness or social isolation were recruited (Table 1). One of the older persons was recruited through another ongoing project where she had expressed loneliness. Three persons were recruited after the researchers had participated and presented the project in a seminar held by a nongovernmental organization (NGO) for persons in retirement, and 2 persons were recruited through contact persons in the municipalities that were included in phase I. They varied in their experiences and proficiency regarding the use of the internet and technology.

Two of the participants were interviewed individually (individual interviews 1 and 2) by the second author and thereafter together in a WS (WS 2 A), along with one researcher and one system developer. Individual interviews were performed since recruitment took longer than expected and only 2 older persons had agreed to participate at the time data collection commenced. The other 4 participants participated in a WS (WS 2 B) together with the system developer and one researcher. Additionally, a research administrator was present at both WSs. Both WS 2 A and WS 2 B were led by the same experienced WS leader who was not part of the research team. The interview and WS questions can be found in Multimedia Appendix 2. During the WSs, the WS leader created a mind map on a whiteboard based on the ongoing discussions in the WSs on a whiteboard for all the participants to see and reflect upon. This was a visualization that was visible to all participants for reflection and served as field notes. The interviews and WSs were audio-recorded. The recorded interviews, as well as the discussions in WS 2 A and B, were transcribed verbatim. Afterwards, the first author read the texts several times. The texts were analyzed by thematic analysis [39]. Meaning units were marked, and codes were noted in the margin of the text. All codes that were relevant to the aim of this phase were combined, and themes were formed. The results of the thematic analysis were compared with the mind maps to identify themes, functions, or other important aspects that were not captured in the mind maps or if the mind maps contained information not found in the thematic analysis, in order to ensure consistency between the participants’ expressed needs in the WSs and in the deeper patterns that emerged from the analysis. The mind maps were thus used as a complementary tool to cross-check and validate the important aspects from the discussion and were reflected in the thematic structure.

### Phase III

In the third and last phase, the purpose was to, in co-design with older people, social service staff, system developers, and researchers, develop a prototype of a health and welfare technology intervention for social interaction to reduce experienced loneliness and social isolation among older people. The older people who were included in phase II were invited to participate in the WSs. Invitations were sent to the 2 municipalities that were involved in phases I and II. One person working as an IT pedagogue for older people who were working in a third municipality contacted the research team, expressing interest in the project, and was also invited to participate. The same experienced WS leader and system developer as in phase II participated in the WSs. The data were collected during the autumn of 2019.

**Figure 2:**
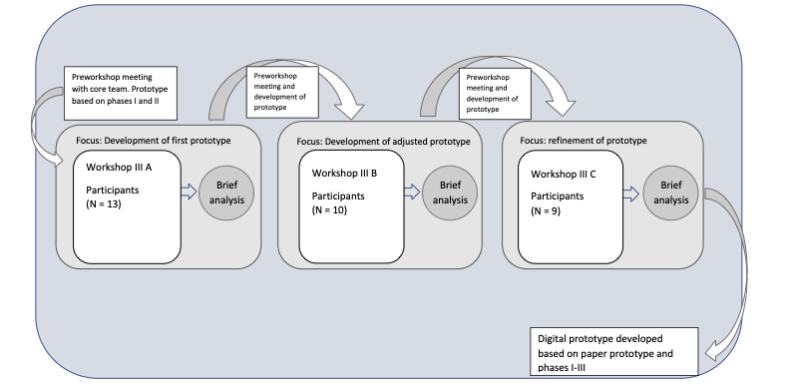
Overview of phase III of the study: 3 participatory co-design workshops conducted in Västerås, Sweden. Participants included older persons with experience of loneliness or social isolation, social service staff, and IT experts. The workshops focused on collaboratively generating, discussing, and refining ideas for the digital solution Fik@ room. The figure illustrates the stepwise procedure, including ideation, prototype development, and feedback iterations (adapted from Hultman et al [40], which is published under Creative Commons Attribution 4.0 International License [41]).

A core team of researchers, along with the WS leader, system developer, and social service administrative staff, conducted pre-WSs before the WSs in phase III, wherein the findings from prior phases or WSs were discussed, and suggestions for a prototype and development of the prototype were made. The first pre-WS discussions took departure from the findings in phases I and II. After WS 3 A, the core team continued the development of the prototype based on the discussions and field notes from the previous WSs, and the improved prototype represented a point of departure in WS 3 B. This was further developed after WS 3 B, and this prototype was used as a point of departure in WS 3 C, wherein the discussions were more focused on details and functions. Figure 2 provides an overview of phase III WS.

Table 1 provides an insight for participants in WS 3 A, WS 3 B, and WS 3 C. In WS 3 B, all of the participants had participated in WS 3 A except for one of the older persons. In WS 3 C, all of the participants also participated in WS 3 B.

The data were collected by audio recordings, to which the researchers and system developers could listen again if something was not clear; for example, if a feature was discussed during the WSs that needed clarification. The audio recordings were transcribed verbatim and used for validating the final version of the prototype. Additionally, field notes were taken during both the WSs and the pre-WS by one of the attending researchers.

### Ethical Considerations

This study followed the research ethics guidelines of the Declaration of Helsinki [42]. An application for ethical review was submitted to the Swedish Ethical Review Authority (Dnr 2019-01870). However, as no sensitive personal data were to be collected, the study design did not fall under the scope of the Act on Ethical Review of Research Involving Humans (SFS 2003:460) [42]. The authority, therefore, granted an exemption from full ethical review and provided an advisory opinion stating that no ethical concerns were identified regarding the study. All participants were provided with information about the study and verbally consented to participate before the data were collected. Older participants were verbally informed by municipal social service staff or the research members at the NGO meetings, as well as in writing through an information letter, and then again verbally through a follow-up telephone call, where informed consent was given. The data were stored to maintain the confidentiality of the participants according to the General Data Protection Regulation. No financial or other compensation was provided to participants for their participation in the study.

## Results

The results are presented for each phase, including a short conclusion for each phase.

### Phase I

#### Overview

The results for phase I are organized by first presenting the results from WS 1 A, including a conclusion, followed by the results of the discussions from WS 1 B, which are summarized for each question discussed. The results in phase I are summarized in a conclusion for both WSs combined. Categories identified in WS 1 A and topics discussed in WS 1 B are summarized in Textbox 1. The notes from the presentations from the 4 groups in WS 1 A were categorized into 3 categories.

**Overview of results from phase I: perceptions of social service staff on how loneliness and social isolation among older people can be reduced by health and welfare technology. Data were collected during 2 workshops (WSs).**

**Categories identified in WS 1 A**

- Identification of intended users
- A digital meeting venue
- Technical advancements

**Topics discussed in WS 1 B**

- What should a digital meeting space for decreasing experienced loneliness contain?
- How could the digital meeting space be implemented?
- Layout aspects and name of the digital meeting space
- How the population could be reached

#### Identification of Intended Users

Participants expressed a wish for a solution that reached persons with home care services and persons in residential care facilities who experienced loneliness. The intervention was discussed as something complementing (but not replacing) human interaction in real life (IRL). It was also discussed as an intervention for those individuals who do not have the ability or possibility to leave their own homes and meet other people due to various conditions, such as immobility or anxiety. This group included people without home care services, but it was also mentioned that these people may be difficult to reach.

#### A Digital Meeting Venue

The intervention was discussed as a digital meeting venue that included activities such as bingo, physical exercise, or games. However, a place for digital meetings where information about activities for the intended group could be found, including a digital calendar in which older people can document upcoming events and which events others will attend, would also be beneficial. The digital meeting space may also include social interaction at any point in time (eg, the possibility of eating together via the web). This type of meeting venue was described as more important than care robots. Nevertheless, the digital meeting venue must be simple to use. Video was mentioned as being a medium for meeting health care personnel and relatives, and could be used for attending larger social events, such as weddings.

A central theme in the discussions was that the digital meeting venue would facilitate new acquaintances with whom the older people could also meet IRL. Dating and building new networks were mentioned. The digital meeting venue could connect people with similar interests and enable them to locate old friends or to discuss existential questions and talk about their lives. This scenario could fulfill the longing for belonging, fellowship, and coherence that was described as something that many older people have.

#### Technical Advancements

It was suggested that a GPS function could be useful for people with dementia, as it could help them leave the house and find their way home. Additionally, alarm systems for falling were discussed as encouraging older people to go outside, which could thereby decrease loneliness and social isolation. Virtual reality (VR) was also discussed as being a factor that could lead to older people having smelling, visual, and hearing experiences that they would otherwise not be able to have. VR could be used for web-based meetings and to visit places. However, VR was also discussed as being very expensive and may lead to economic exclusion. The use of TV screens for planning and controlling daily schedules and as a tool involving speech synthesis to support people with impaired visual and hearing functions. The needs were discussed as differing between men and women.

#### Conclusions From WS 1 A

All groups discussed some type of digital meeting venue for older people, with the possibility of meeting physically after an initial digital contact, as well as a meeting venue for older people to tell stories and be able to find both new and old friends. This meeting venue could be accessed from home and complements human interaction IRL. A digital meeting venue was the point of departure for WS 1 B.

#### The Content of a Digital Meeting Venue for Decreasing Experienced Loneliness and Social Isolation

The digital meeting venue could be built with rooms for groups to meet in for activities such as bingo, quizzes, online games, physical exercises, or church masses (both live and recorded). It may also include lectures, opportunities for text-based chats, matchmaking, reading, collaborative cooking, movies, study circles, craft groups or book circles or singing together. It was also suggested that there should be separate rooms available for men. Fixed time for digital meetings and weekly programs was suggested, as was the possibility of seeing who was available online.

It was also discussed to include professional support to help make plans for the future, and a suggestion involved including links for future meeting times to family counseling, legal counseling, and housing agency support. The meeting venue was suggested to include local, national, and international news relevant to the users. In digital meeting venues, people who experience social isolation due to physical restraints may have the opportunity to meet others and participate.

#### Strategies to Implement a Digital Meeting Venue

Digital literacy was expressed as being crucial among both staff and users for successful implementation. It was further discussed as to whether it should be an individual responsibility to have the necessary equipment to participate in the digital meeting venue or if it should be provided to the intended users. Ownership of the digital meeting venue may be for the municipality or NGO. Links to technical support should be available at all times, where NGOs may be a resource.

The digital meeting venue must be a safe place. Security may be obtained by using a log-in procedure. Young people may experience that using social media affects their mental health, and this scenario should also be considered among older people when implementing digital meeting venues.

#### Layout Aspects and Name of the Digital Meeting Venue

The staff expressed that the digital meeting venue should have a pleasing and appealing design to arouse curiosity. When considering the intended target population, it should be easy to use and have contrasting colors, few, and large buttons. Speech synthesis was preferred. The digital meeting venue should be able to be used on both tablets and computers, with information available in several languages. It was also discussed that VR glasses and gloves would be interesting options to include. A personal page should also be available.

Several names were discussed to reflect the imagined benefit of the meeting venue: “Atle,” as the ice-breaker boat; “Simul,” as Latin for social interaction; and “the golden middle age,” “it said click,” “mature dating,” “senior spot/app/channel,” as the digital meeting space. The name of this program should not be associated with loneliness and aging. It was also suggested that the intended target group should choose the name of the web-based platform.

#### Strategies to Reach Intended Users

Several strategies to distribute information were discussed, such as flyers to distribute in primary care (as part of the customary information to all people older than 75 years), advertisements in local newspapers, on NGOs and municipality web pages, on Swedish health care direct (1177), and at gyms and churches where the target group can be contacted.

#### Conclusion Phase I

The conclusion from WS 1 A and WS 1 B was that the focus for further research should be on a digital meeting venue where older people can meet online for various activities that also include information directed to older people’s specific life situations. The program should also be easy and safe to use. The intended users would include older people experiencing loneliness or social isolation, which could be identified by staff within several occupations and fields. The meeting venue should facilitate the discovery of both new and old friends, and the name may indicate this effect (but should be chosen by the target group).

### Phase II

#### Overview

In phase II, the thematic analysis resulted in 6 themes reflecting the expressed needs related to loneliness and social isolation, as well as how these needs may be targeted by using health and welfare technology (Textbox 2). In Figures S1 and S2 in Multimedia Appendix 3, translated mind maps from WS 2 A and B are provided. Quotations from participants are included to illustrate and support the findings.

**Overview of the themes identified in phase II: needs and preferences related to loneliness and social isolation perceived by older people. Data were collected through 2 semistructured interviews and 2 workshops.**

**Themes**

- A gateway for finding new acquaintances
- Provide opportunity for both light-hearted and deeper conversations
- Being part of a community
- Enabling new ways for older people with barriers to interaction
- Available, safe, and varied forms of social interaction at any time
- An easy-to-use and appealing web-based platform

#### A Gateway for Finding New Acquaintances

A central theme in the participants’ descriptions of loneliness was the challenge of finding new acquaintances. This was seen as crucial for reducing feelings of loneliness but increasingly difficult with age. In earlier life stages, especially before retirement, new social connections often emerged naturally, for example, through work. After retirement, such contexts disappeared. Additionally, aging was associated with losing friends and spouses, which led to a shrinking social network and reduced opportunities to meet new people.

But then… then, what I’m experiencing now is that several friends… you lose them, they die, they die… and it feels a bit… uh, sad, and it has actually hit me very hard because they are close friends. So in that sense, loneliness strikes. But… but at the same time, I also see opportunities because I’m still curious about life and want to be part of it.

participant WS 2 B

Participants expressed that finding new acquaintances required initiative and courage, especially IRL. Activities such as walking with others or meeting people during daily walks were mentioned as potential methods for making contact. However, it was often difficult to find such groups or to approach strangers. In this context, web-based interaction emerged as both a barrier and an opportunity. Digital tools were often used to maintain existing relationships, but seldom for meeting new people. Still, participants saw potential in digital platforms for enabling initial connections that could lead to in-person meetings later.

It's like this - most people, if we put it that way, might start with something digital. But in the long run, it's kind of like a dating site - you start there, but eventually, you still want to meet in real life.

participant individual interview 2

Participants emphasized that initiating new conversations via the web should feel natural, but few platforms were known to support this. Existing digital communication was typically limited to known contacts, and reaching out to someone new, especially without a clear reason, felt awkward or even inappropriate.

If you have the contact, you can maintain it. But getting a new contact, I think that's difficult.

participant WS 2 A

The internet was seen as a tool for finding others with similar, or even rare, interests, but this was often difficult both digitally and IRL. The lack of accessible, interest-based meeting spaces contributed to a sense of isolation for those whose hobbies or passions were not widely shared. There was also a clear longing among some participants for deeper friendships and meaningful new connections, even with people who had different backgrounds or interests, or from the opposite sex. This was seen as both desirable and particularly challenging.

There has to be something that makes people meet, you know. You need a reason to start talking, maybe several times. The first time, nothing really happens. It’s hard to get to that point.

participant WS 2 B

To facilitate new connections, the participants suggested the creation of a digital “gateway”—a platform where people experiencing loneliness could meet regularly via the web. This would allow for repeated interactions and the gradual building of trust, which could eventually lead to offline friendships.

So, some kind of catalyst is needed to get these kind of discussions started.

participant WS 2 B

#### Provide Opportunity for Both Light-Hearted and Deeper Conversations

The participants expressed a need for a digital platform that provided opportunities for different types of encounters, such as a meeting place for light-hearted conversations and a place to have fun together to find joy in both fellowship and conversations, but also as a place for deeper conversations about various subjects, such as existential matters. Deeper conversations were expressed as being the one topic that provides meaning to social encounters. The ability to express significant feelings to others when alone was considered very important, not only to be listened to, but also to listen to others.

[...] I didn’t say a word, and she said, ‘You were very nice to talk to,’ and I hadn’t said anything”. “You were listening, and that’s what’s needed.

2 participants in WS 2 B

However, it was also expressed as being important that a digital platform should also provide the opportunity not only to talk about difficult matters but also to find people to laugh with and joke about things together. Some of the participants stated that it took some time to express their true selves and how they could contribute to a conversation, and sometimes it could be easier to write in a chat.

That there are those, I’m the kind of person, who wants a slightly deeper conversation. But then there are others who don’t want that, and both need to exist, I think.

participant WS 2 A

The digital platform should also provide an opportunity to talk about special interests, such as specific subjects that one has deep knowledge about and may have worked with earlier in life.

It was also expressed that they desired to help others in similar situations and wanted to share these experiences with others. It was considered desirable to be both able to give advice and provide support for others, as well as to find support and guidance for oneself. Being important to others was expressed as valuable and could make one feel significant to others.

Yes, I mean, [...] I’m used to being a widow and knows how… one is treated and how… how friends react when you are a widow. Then it can be good to be able to guide someone else who has just become a widow, that’s how I see it.

participant WS 2 B

The digital platform was discussed as being something similar to the breakrooms from former workplaces or, as one participant referred to, a digital smoking room that was described as the place where “everything happened,” with a special fellowship for those who attended these locations.

Yeah, but that sense of community, the new community, it was very much built in the smoking room, as I said, because that was how it was back then.

participant WS 2 A

The participants missed the natural context at work for meetings such as those that occurred in the breakroom. In retirement, the participants described experiencing involuntary exclusion. In the breakroom at work, one could simply enter, sit down at any table, and discuss whatever topic they wished to discuss, sometimes light-hearted, sometimes deeper conversations, or sometimes about specific interests with like-minded individuals.

When in retirement, it was described as more difficult to meet new people for conversations, as one may have previously done in the breakroom. However, no individual enters an empty breakroom; there must be people in the breakroom to talk to. In a web-based setting, it was expressed that it is important that there is a maximum number of the people allowed in this setting, as too many people in a digital format would make it difficult to communicate without disrupting each other. It was also suggested that someone should be responsible for the conversations that occurred. In the breakrooms at work, there were discussions about important things, and agreements on a specific topic were not necessary, which resulted in stimulating conversations. To gather more people in one location could stimulate the initiation of those kinds of conversations.

It was also expressed that those participating in web-based meetings should share similar experiences of loneliness, and that no professionals should be involved.

When it comes to loneliness, I think it's better to be in groups where people themselves have experience of what is being discussed, so no professionals.

participant WS 2 B

#### Being Part of a Community

Participants described how retirement often leads to a loss of the natural social interactions that come with working life. Without the daily structure of colleagues, meetings, and casual conversations, many older persons experienced a shift from being active participants in a community to becoming observers from the sidelines. This change was perceived as contributing to feelings of loneliness and exclusion. One participant reflected poignantly on this transition:

Well, when you get as old as I am now, you feel there's a kind of boundary, and I notice it with other friends who are the same age. They think seventy is some kind of threshold. It's a bit of both, you're on the side now because you don't work anymore. You visit your children and grandchildren, and then there are other young families nearby, but you are on the side. And that's a bit sad.

participant WS 2 B

This experience of being “on the side” highlights the social rupture that retirement can bring, underscoring the need for new ways to maintain a sense of community and meaningful involvement later in life.

The internet was described as a resource for finding information on where to seek help when feeling lonely, as well as for sharing information and links with others. Simply seeing other people, whether online or offline, could help a person experiencing loneliness feel a sense of belonging.

It's just like that, in some way, that there are people around you.

participant individual interview 1

This scenario could occur during a walk or while being online. For example, it may be appealing to attend a concert alone, but it does not automatically lead to feelings of belonging. Loneliness was described as being alone with no opportunities to share with or talk to others. The participants expressed that different people may have different perceptions of what makes one feel lonely; however, Swedish culture was described as being a factor that contributes to older people feeling lonely, and men were described as being particularly vulnerable.

Well, our immigrants, they socialize much, much more within the family and among relatives. So, they are not alone in the same way we are.

participant WS 2 B

Neighbors and community members who look out for one another were highlighted as important for people experiencing loneliness. Having no one to talk to for more than a day was described as threatening to one’s well-being. However, the encounters needed to counteract loneliness did not have to be significant; even brief conversations, for example, with home care staff, could help alleviate the feeling.

But I notice that when I'm alone - well, one day is fine, but after two days or so, I go downhill. So... meeting someone, and it doesn't have to be anything special really, just talking a little - not just about the weather either, but something a little deeper - then you feel better afterwards. At least I do. So, I'm probably more social than I thought. Yes. And when you were working, it was so natural, meeting people, and for me who had students and so on, it could be nice to come home and have it be quiet.

participant WS 2 A

The participants also spoke about the importance of connecting with like-minded individuals; people who share similar levels of knowledge and interests.

Among like-minded people, they should have roughly the same knowledge. My colleagues had a certain way of understanding things, and that's the kind of discussion you move within.

participant WS 2 B

Finally, participants noted the challenges of sharing personal interests that others may not understand.

It's hard for me to get people in general to understand my interest. What it is about. Many just say, 'That's not something I'm into.'

participant Individual interview 2

Finding like-minded individuals was thus important for feeling part of a community.

#### Enabling New Ways for Older People With Barriers to Interact

Meeting digitally was described as an important opportunity for older individuals facing barriers that make it difficult to leave their homes. These barriers included a lack of initiative, physical limitations, medical conditions, or being naturally reserved or shy.

I can't attend courses because there's air conditioning, and I can't go to the movies, I can't take the bus, train, or fly... So, I become very lonely.

participant WS 2 B

Web-based activities, such as playing games or cards, were described as tools for reducing loneliness. These games could be both synchronous (played together in real-time) or asynchronous (played over a longer period), offering flexible ways to stay socially engaged. Although playing alone could provide a temporary distraction from feelings of loneliness, participants emphasized that digital interaction with others was more valuable for their sense of connection.

But for example, if you say that many people who are lonely also have difficulty moving and end up sitting alone at home. Then, instead of meeting in person, you could have a conversation in a group like that.

participant individual interview 1

#### Available, Safe, and Varied Forms of Social Interaction at Any Time

Participants described the emotional highs and lows associated with loneliness. When the telephone rang, it could initially spark excitement, a hope that something meaningful was about to happen. However, this feeling often turned to disappointment, for example, when the caller turned out to be a salesman. Such experiences could reinforce feelings of loneliness and lead to further withdrawal from social interactions. One participant shared an experience of reaching out to a friend during a difficult time, only to be rejected. This highlighted a broader need expressed by the participants: to have access to someone to talk to when feelings of loneliness become overwhelming and urgent.

Yesterday, I needed someone to talk to, so I wrote to a friend. ‘I’m watching [a TV-show], can I get back to you tomorrow morning?’ That was the response. By then, it was too late.

participant individual interview 2

At the same time, concerns were raised about using the internet for social connections. Participants discussed the risks of being tricked or swindled via web and emphasized the importance of safe, trustworthy digital platforms.

I'm bad with Facebook and bank passwords because my account was hacked during my first visit. I became very, very scared. Neither were the police interested, and I didn't know what to do. And I got really scared.

participant WS 2 B

A key aspect of feeling secure was being able to see who one is communicating with, such as through video calls. Visual contact not only fostered trust but also made the interaction feel more genuine. However, participants also acknowledged that speaking could sometimes feel too demanding, and that writing, via e-mail or chat, could be a gentler way to reach out when feeling vulnerable. That said, while writing could offer a temporary outlet, participants noted that chatting alone did not significantly reduce feelings of loneliness, and direct interaction remained crucial for emotional connection.

#### An Easy-to-Use and Appealing Web-Based Platform

All participants had experience in using digital tools; however, some of them preferred to use the telephone, which was a method that they were more accustomed to. They often used the telephone to decrease feelings of loneliness. Facebook was also used, mostly for reading what others had published. Some of the participants had computers that they did not use and perceived themselves to lack knowledge about how to use them. Others used computers every day. Curiosity and willingness to try new things were described as being important for learning about digital tools. The fact that older people often lack the knowledge to use digital tools, as well as the need for support to use digital tools, was discussed as being important when designing digital tools for older people. The tool itself should be simple to use and easy to obtain, and the layout and included images must fit the intended population. Images were preferred over long texts.

Well, as I said, I think it should be easily accessible and clearly laid out.

participant individual interview 2

The design should also accommodate the specific needs of older people with disabilities.

I have some difficulty writing, I'm shaky, I have an illness, so I find it hard to press the... buttons, quite simply. Does that fit in here somehow?

participant WS 2 B

#### Conclusion Phase II

The analysis of the recorded material, together with the mind maps, formed the basis for the development of the first prototype of a web-based platform with functions that captured the expressed needs of the participants in phase II.

The web-based platform should be designed to enable people to find new acquaintances and act as a gateway to reach people with the same (but also different) interests. The web-based platform should enable both light-hearted and deeper conversations, with the ability to help each other in difficult situations and share experiences. However, the participants did not want any “professionals” to participate on the web-based platform. The web-based platform was described as desired to be similar to the breakroom at work in one of the WSs. One advantage of a web-based platform is that it enables older people, who sometimes have barriers to leaving their apartments to participate, to interact with others, as well as the fact that interactions are available at any time. However, having scheduled time for meetings was suggested to be optimal. It was important that the web-based platform was safe to use and provided different ways to communicate, but also be easy to use and appealing to the intended users. Options of continuing the relationships outside of the web-based platform and to “make appointments” were discussed in both WSs. The mind maps, together with the identified needs from the thematic analysis, were the basis for the first prototype presented in WS 3 A in phase III.

The name of the web-based platform was also determined. We did not decide on “the smoking room” option that was suggested; instead, “the Fik@ room” was chosen as the name for the web-based platform, as “fika” is a Swedish word for having a break (preferably together with others), while having a cup of coffee or tea.

### Phase III

#### Overview

**Figure 3:**
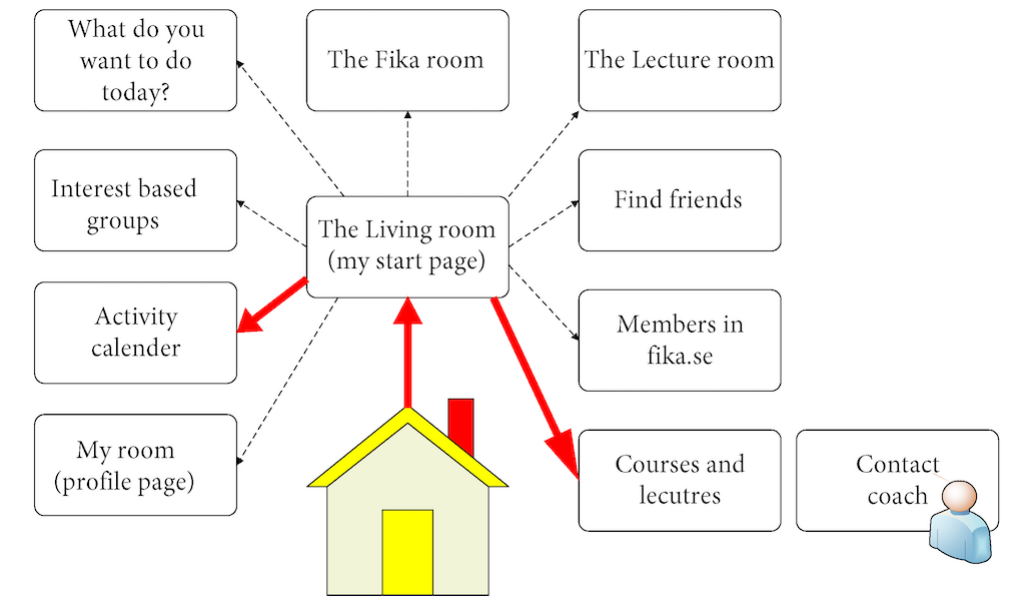
Prototype 1, translated from Swedish, of the web-based platform presented and discussed in workshop 3 A (phase III). The workshop was conducted with older persons with experience of loneliness or social isolation, social service staff, and IT experts. The figure illustrates the first version of an overview of the Fik@ room prototype, including the digital break. Feedback from the participants informed the subsequent development of the prototype.

**Figure 4:**
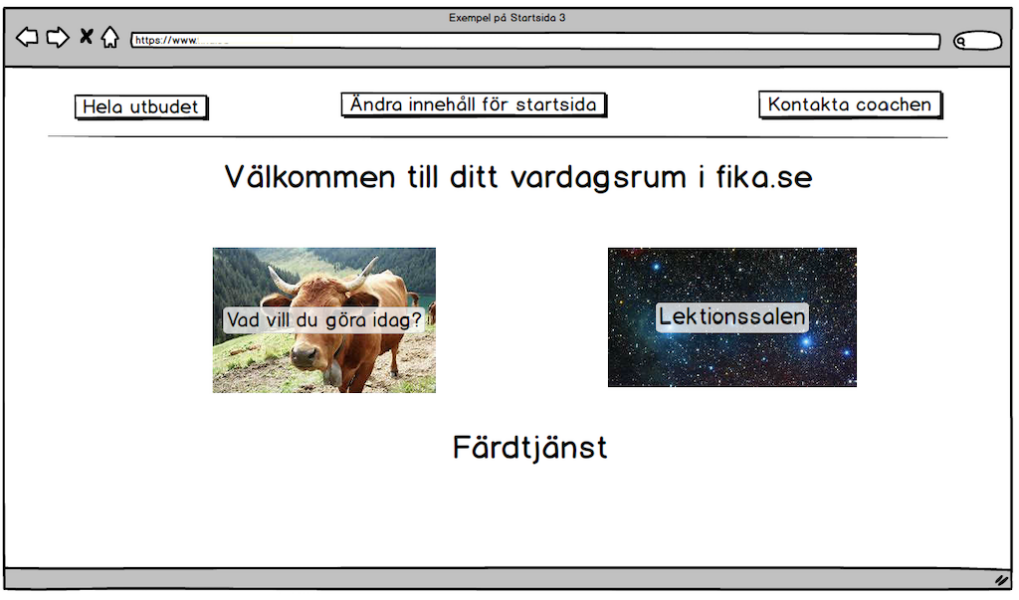
Example screen from prototype 1 of the Fik@ room, showing ”Your Living Room” (Ditt vardagsrum). This screen was presented and discussed during workshop 3 A (phase III) with older persons, social service staff, and IT experts. ”The Living Room” provided links to “What do you want to do today?” (Vad vill du göra idag?), ”the Lecture room” (Lektionssalen) and a link to ”External transportation service” (Färdtjänst). Feedback from participants on the usability and relevance of this design informed later iterations of the prototype design.

In the first pre-WS meeting, before the first WS in phase III, a digital prototype (prototype 1) was developed based on the analysis of the transcribed interviews and WS recordings from phases I and II, as well as the mind maps from phase II (Figure 3). Figuratively, prototype 1 was built as a house with different rooms where different activities occurred, and was the point of departure for WS 3 A. All of the different boxes in Figure 3 were operationalized as a web page/screen. One example is provided in Figure 4, which shows the living room that was the starting page from which the different functions or rooms could be reached. This user exemplified in Figure 4 had chosen to have “What do you want to do today” and the “Lecture room” as shortcuts from the Living room.

#### Results of WS 3 A and the Core Team Meeting in the Pre-WS Meetings

The topics brought to discussion by the WS 3 A participants can be found in Textbox 3.

**Topics brought to discussion by the workshop 3 A participants.**

- Need for a digital coach as part of the web platform.
- There is a need for general support for using web-based platforms.
- Provision of information on what activities are occurring in the community for the target population.
- Distribution of software and hardware for older persons.
- A web-based platform should be easy to use, and all functions and rooms in prototype 1 could be difficult to navigate. The web-based platform should be stable and not change over time in terms of functions.
- Web-based platforms could be connected to other important functions and organizations of society, such as transportation services.
- Some type of calendar to find activities for older persons.
- Security of and access to the web-based platform, and a wish that all persons should be in attendance on equal terms.
- How can we connect with and find new acquaintances, such as based on geographical factors or by interests?
- Fixed appointments in time for joining the digital platform.

In the pre-WS meeting before WS 3 B, the core team members had an impression that prototype 1 may have been too extensive for the participants to grasp, and that the discussion mainly focused on support for using the web-based platform. It was subsequently decided to scale down the functions of the web-based platform, but to retain functions that could still meet the expressed needs. Therefore, Prototype 2 included the following functions: a start page with coffee tables as a central function that supported different preferences for communication (chat, audio, or video conversations); a profile page; a bulletin board; and a support and information function, as the point of departure for WS 3 B. The topics brought to discussion by the WS 3 B participants can be found in Textbox 4.

**Topics brought to discussion by the workshop 3 B participants.**

- The support function for technical issues or if someone misbehaves. Ethical principles, such as a code of conduct, could be stated. Instructions could be included in video format. One page for support and instructions was suggested.
- The profile page could have optional choices of photo and text for the description of interests. It should be possible to see who else is online.
- Fixed appointments in time for “fika” could increase the possibility of meeting if a few individuals are online/use the digital platform. However, the web platform should be open 24 hours a day.
- The overview of the coffee tables should include who has joined the tables and what topics are discussed at the tables. It should also be simple to join and leave tables if the topic is not interesting. The individual who starts the table can pick a topic and form for communication (chat, audio, or video conversations).
- The bulletin board should be a place for advertising activities such as meetings on the web-based platform (such as specific topics), in real life, or general notes with tips. Topics need to be relevant, and no old posts should be kept. Contact information for the individual who posted and a link to that person’s profile page should also be provided.
- The suggested activity calendar could include the possibility of inviting others to events, both online on the web-based platform and in real life, as well as seeing what other events will be attended by others. Herein, it could be a dedicated page for local activities from the municipality and nongovernmental organizations to be advertised.
- The design must be simple, and the web-based platform must be easy to use and navigate, as well as not be intimidating. There should not be extensive functions and information.
- Security issues, such as the application of the General Data Protection Regulation and access to the web-based platform, were discussed.
- There was a request for private spaces for deep conversations. These places/spaces could be advertised on the bulletin board. These “rooms” could be used for structured meetings and to follow up on topics such as existential questions with “homework.”
- The possibility of sending messages (both private and more official) was requested.

In the pre-WS before WS 3 C, minor adjustments were made to Prototype 2 with a focus on the functions discussed in WS 3 B. The discussions in WS 3 B were perceived as being more focused on the functions; moreover, due to the fact that it was discussed how the web-based platform should be easy to use and navigate, it was decided to have the coffee tables (the fika tables) as a central function and start page. Additionally, due to the fact that the activity calendar may be difficult to use and expensive to develop, it was not included in Prototype 3. Prototype 3 included profile pages, a bulletin board, a support function, an information page, and a page with links to important societal functions. During WS 3 C, a discussion of whether some functions were lost and whether some functions would be tested, such as how to write messages on the bulletin board and how to conduct a video conversation, should be performed. The topics brought to discussion by the WS 3 C participants can be found in Textbox 5.

**Topics brought to discussion by the workshop 3 C participants.**

- An overview of online users. Additionally, the possibility of having a “friend-list” and seeing who is online.
- Profile picture should be used to identify who sits at the tables.
- A reasonable number of participants should be 4 individuals at one table.
- The design must support the use of aids for those with loss of function, such as hearing or vision impairments.
- If many people are online, how can we group them; specifically, should there be one fika room for each municipality? Will this be a national app or a regional app?
- Fixed times for fika sessions each day could help people to log in at the same time. Many people must be logged in for the web platform to work.
- One table is ready to use when logging in to make it easy to start.
- A table overview was suggested, in order for individuals to see who is at the other tables.
- Empty tables should be removed.
- The participants were positive about the video meeting test.
- A suggestion to be able to share screens in meetings was expressed.
- Rooms/tables for private conversations were suggested. Additionally, it should be possible to send private messages.
- The need for technical support.
- The possibility of inviting other people to use the platform.
- The advertisements on the bulletin board must be up to date. There should also be a possibility to answer advertisements and see who has answered them. Advertisements should be sorted by topics or how to meet, digital or in real life.
- Pages with links that are important for an older person experiencing loneliness.

#### Conclusion Phase III

The 3 phases resulted in the development of a paper prototype of a web-based platform that was named the Fik@ room, aiming at reducing experienced loneliness and social isolation. The main function of the Fik@ room involves coffee tables where people can interact similarly to how breakrooms functioned at work before retirement. Participants had discussed in phase II examples of separate groups, such as for men or widows. This was interpreted as an indication of the importance of enabling users to choose with whom they interact. In the prototype, digital coffee tables can therefore be arranged for specific groups (eg, “men only” or other interest-based discussions) if users wish, and such tables can also be announced on the bulletin board or labeled with the theme of the discussion.

One important finding was that the web-based platform must be easy to use to not exclude older people with lower digital literacy. Therefore, a prioritization among the functions from Prototype 1 was developed for Prototype 2. The needs expressed in phase II were still perceived as being met (to a large extent) in the final prototype. Except for the coffee tables, the main functions in Prototype 2 were the bulletin board, the profile page, a support and information page, and a page for relevant links for the target group.

The need to meet new people, as well as to identify old acquaintances, has both lighthearted and deeper conversations, and find people for activities IRL, in addition to the other needs that were expressed, could be fulfilled by the Fik@ room.

In phase I, one finding was that the web-based platform could include different activities and professionals. In phase II, activities were also mentioned, both of which can occur on the platform and IRL. The more prominent need to keep the platform simple, easy, and intuitive to use was nevertheless prioritized. Herein, the bulletin board may play an important role, and instructions will be developed regarding how the bulletin board may be used for arranging meetings both inside and outside of the platform. The log-in procedures, as well as who has access to the Fik@ room, will enable a secure and safe platform.

In Multimedia Appendix 4, a URL to one of the developed instruction videos regarding how to navigate in the Fik@ room is included. This video was also included in the final design of the application and used during the feasibility and pilot studies. It shows the final visual interface of the Fik@ room [43].

## Discussion

### Principal Results

This study describes the iterative design process that resulted in the application of the Fik@ room for older persons experiencing loneliness or social isolation through participatory design procedures with different key stakeholders. The main result of the study is the production of the paper prototype version of the Fik@ room, supporting social interaction among people experiencing loneliness or social isolation. The name of the application (the Fik@ room) is a direct translation of the Swedish suggestion “Fikarummet,” which was proposed by participants in the WSs in 2019. The term “fika” is the Swedish word for having coffee/tea, taking a break, and being socially interactive, which represents a common phenomenon in Swedish workplaces. The letter “a” was replaced by @ as a symbol for the digital form of this fika room. The participants in the WSs drew a parallel to the coffee/break room at their former working place, where they would sit down at a table and were socially interactive by having conversations with their colleagues. They missed this fika break from their working life and wanted the web-based platform to fill this gap. The results from the different phases contributed to fulfilling this need in the Fik@ room. The developed prototype was designed to primarily target social rather than emotional loneliness [44] as the latter is challenging to address through digital solutions only. The platform is designed to offer opportunities for initial contact and social interaction, allowing users to meet new people and, if they wish, continue interacting with the same individuals over time. As expressed by the participants, such a platform may serve as a starting point for developing relationships that could later transition to face-to-face meetings or other communication channels. The platform should therefore be regarded as a complement rather than a substitute for in-person social interaction.

### Comparison With Prior Work

General successful interventions for decreasing loneliness and social isolation among older people include one-to-one or group interventions, as well as community activities, that are performed as both face-to-face and technical interventions [27]. A recent umbrella review showed that ICT interventions can be effective at increasing social support, increasing contact with others, and increasing self-confidence [26]. Although many of the successful interventions for social connectedness that are presented in the reviews are based on communication with family and friends, the effectiveness of developing new friendships needs to be focused on in future studies, such as this study. The use of the Fik@ room does not require an established network of contacts and can also be used at any time, as long as others are logged into the app. Of course, this implies that there must be a critical minimum number of users of the Fik@ room, which should be considered when implementing the application of the Fik@ room. Otherwise, an empty Fik@ room may contribute to feelings of loneliness and social isolation.

The Fik@ room was evaluated in a feasibility study [45] and a pilot study [46], and the implementation of the Fik@ room was evaluated from an organizational perspective [47]. The web-based platform has been shown to provide older persons with new contacts and reduce experienced loneliness, thus serving as a complement to other relationships and activities [45,46]. It has been shown to be feasible to develop new relationships and thereby be suitable for those who lack family and friends. Moreover, over time, relationships tended to undergo a transformation into deepened conversations among friends [45]. However, it may not be suitable for all individuals. Not all individuals appreciate meeting and getting to know new people, especially not in a web-based setting. A prerequisite for using the web-based platform involved motivation and a perceived need to meet new acquaintances via the web [46]. In earlier studies, it has been shown that web-based social activities have the potential to improve older people’s social contacts and increase participation in activities, which can reduce their experiences of loneliness [48].

The web-based platform was designed to be easy to use, which has been confirmed [46] and may have been achieved through the involvement of key stakeholders [30]. However, some of the older persons considered digital technology devices to be too complicated to use due to joint pain in their hands or because touchscreens do not respond to older people’s dry fingertips. Hence, the use of a web-based platform on a personal computer instead of a tablet should be optional. The development of new technology could preferably include a dictation function as an option for writing, and this was planned to be used in the chat function in the Fik@ room. Moreover, as we experienced, a lack of digital confidence and skills may be a barrier [47]. Providing continuous support with login advice and handling the different features of the web-based platform could overcome these barriers.

Regardless of how good the product is, it may be difficult to reach older people in need of social interaction. According to the abovementioned studies, care professionals and social services personnel may be the appropriate people for recruiting and introducing the intended target group [45,46], thereby increasing the chances of reaching potential users.

The Fik@ room was developed in co-design, with the notion that by involving older persons and the staff who meet these persons every day in the design, the web-based platform would be tailored to the end users’ needs, thereby being easier to implement. Experts within the field, such as researchers and system developers, were also involved in contributing to broadening the perspective to find new solutions. The implementation of interventions is complex and is, therefore, important to consider during the development process. In addition to recruitment and being able to reach the intended target group, personnel must be able to introduce the web-based platform [47]. This indicates that they will need knowledge about the web-based platform and be able to show it to the intended user. If not perceived as being a useful tool by personnel, they will be reluctant to introduce it to older people. To feel comfortable introducing digital products, management will have to increase time and costs to improve digital skills among personnel, thus allowing for time to use and become comfortable with the product. Moreover, there is a need for IT support. It has been shown that implementation involves an entire organization (to varying degrees) and that all levels need to be involved to create the necessary conditions for successful implementation. Future work will also need to address implementation strategies for reaching a critical minimum number of users to ensure meaningful engagement. This may include partnerships with NGOs, community organizations, and municipal services to facilitate user onboarding. In the feasibility and pilot study, a suggested time for “fika” was communicated to the users in order to ensure a critical minimum of users [45,46].

### Strengths and Limitations

The strengths of this study included the involvement of several different key stakeholders in the development of the Fik@ room. To better understand users’ needs, creating an intervention that is engaging and easy to use is suggested to have the involvement of key stakeholders in development, and should be a standard procedure [30]. The valuable input from different sources may have strongly contributed to the successful implementation of the Fik@ room presented in other studies [46], and the issues during implementation were not associated with the use of the Fik@ room but with other aspects [47]. The co-design included key stakeholders and experts from several fields and in different phases of development, thus contributing to a broad base of knowledge in the development process.

A potential limitation of our approach is that the process started with WSs with social service staff, which may have implicitly framed the project and the input from the older persons in phase II. Even though phases I and II were conducted and presented in chronological order, they should be regarded as parallel co-design events rather than sequential phases building on one another, to put the needs of older people first and in line with Robert’s co-design model [37]. To minimize this influence, the interview guide used in phase II was designed to explore older people’s experience and preferences broadly, rather than being based on the discussion in phase I. In addition, different members of the research team collected data in these 2 phases, which further minimized the risk of bias. Nevertheless, the focus on a digital solution was predefined in this study, which may have hindered the participant in all phases from expressing needs for a nondigital solution. It is also possible that the older persons who consented to participate were those who were already positively inclined toward digital solutions.

At some points, we obtained conflicting suggestions in the phases and from different participants. Recommendations suggest that co-design studies be conducted in small, frequent consultations with stakeholders to understand their preferences [49]; by the time of the last WSs, it was perceived that all of the participants agreed on the final prototype. One further limitation of the study was the small sample size and representation within the different groups of key stakeholders. There was only one man who participated (he represented the intended target group), and there were also small variations regarding cultural background among the participants. The small sample size and the homogeneity of the sample may have contributed to the final design not being sufficiently designed for other groups that were not included in this study. Recruitment for this study was challenging, and participation of older persons was limited to 1 man and 4 women, all ethnically Swedish and born in Sweden. This homogeneity restricts the transferability of our findings, particularly regarding potential cultural differences in experiences of loneliness and social isolation. Future studies and iterations of the Fik@ room should strive to include underrepresented groups, such as more men and individuals from immigrant communities, to better capture diverse perspectives and address potential cultural barriers. Regarding sample size, there is no consensus regarding the group size on co-design development studies [30], and the studies evaluating the Fik@ room [45,46] did not demonstrate that it was not gender or culturally inappropriate. Another limitation was that the participants in phase I opposed being recorded, which may have influenced the depth of the data analysis in phase I.

### Conclusions

The study process led to the development of the prototype of the Fik@ room, which is a digital venue for older people experiencing loneliness or social isolation to meet and interact with others with the same experiences. The features of the Fik@ room are based on the needs expressed by social service staff with professional experience working with this group and older people with first-hand experience of loneliness or social isolation. The central aspects that were included in the development and the final digital prototype were an easy-to-use application (meaning a program including a few functions and intuitive use). The layout and colors should focus on being appealing to an older population. The main features include a digital fika room (break room) with tables where the user of the Fik@ room can “sit down” for a chat, an advertisement board for announcing meetings in or outside the Fik@ room, and a profile page for each user. Only persons experiencing loneliness and social isolation should have access to the Fik@ room. The development of the Fik@ room led to an application that was perceived as being easy to use and that provided the opportunity for new contacts, thus implying that the development was successful when implemented with support for users.

## Appendix

Multimedia Appendix 1 COREQ Checklist.

Multimedia Appendix 2 Interview and workshop gudie phase II.

Multimedia Appendix 3 Mind maps developed during WS in phase II (Figure S1 and Figure S2).

Multimedia Appendix 4 URL to one of the developed instruction videos regarding how to navigate in the Fik@ room. This video was also included in the final design of the application and used during the feasibility and pilot studies and shows the final visual interface of the Fik@ room.

## Abbreviations

COREQ: Consolidated Criteria for Reporting Qualitative Research
IRL: in real life
ICT: information and communication technology
NGO: nongovernmental organization
VR: virtual reality
WS: workshop

## Data Availability

The datasets generated and analyzed during this study are not publicly available because data sharing was not included in the agreement with participants. Datasets are available from the corresponding author upon reasonable request.
