# Supplementary material for: Co-Designing a Digital Solution for Decreasing Loneliness and Social Isolation Among Older People in Sweden: Explorative Study
Source: JMIR Form Res. 2025 Nov 21;9:e78213. doi: 10.2196/78213 (PMC12680934; doi:10.2196/78213)
Supplement: Multimedia Appendix 1 [file formative_v9i1e78213_app1.pdf]

## COREQ Checklist

| Topic | Item No. | Clarification of item | Presented in manuscript or not applicable (N/A) |
|---|---|---|---|
| **Domain 1: Research team and reflexivity** | | | |
| *Personal characteristics* | | | |
| Interviewer/ facilitator | 1 | Which author/s conducted the interview or focus group? | WSs and interviews were conducted by authors and an experienced WS leader: phase I authors three and four; phase II interviews author two; phase II WSs author one; phase III WSs author one and two(Methods section) |
| Credentials | 2 | What were the researcher's credentials? E.g. PhD, MD | All authors are PhD-level researchers (Author contributions section) |
| Occupation | 3 | What was their occupation at the time of the study? | Researchers were employed by Mälardalen University (Author contributions section) |
| Gender | 4 | Was the researcher male or female? | All researchers were female |
| Experience and training | 5 | What experience or training did the researcher have? | All researchers/authors had prior experience in qualitative methodology and conducting interviews (Methods section) |
| *Relationship with participants* | | | |
| Relationship established | 6 | Was a relationship established prior to study commencement? | Relationships were established through prior projects and NGO meetings (Phase II). No prior relationship in phase I. Participants in phase III had all been involved in phase I or II except one who contacted the researchers by own interest (Methods section) |

| | | | |
|---|---|---|---|
| Participant knowledge of the interviewer | 7 | What did the participants know about the researcher? e.g. personal goals, reasons for doing the research | Participants were informed about the study and its purpose described in information letter and all had opportunities to ask questions (Ethics section) |
| Interviewer characteristics | 8 | What characteristics were reported about the interviewer/ facilitator? | Researchers had experience and interest in qualitative research and digital interventions. The WS leader had previous experience and training in leading co-design WSs (Methods section and Author contributions) |
| **Domain 2: Study design** | | | |
| *Theoretical framework* | | | |
| Methodological orientation and theory | 9 | What methodological orientation was stated to underpin the study? e.g. grounded theory, discourse analysis, ethnography, phenomenology, content analysis | Participatory research design based on experience-based co-design model. Thematic analysis including its epistemological underpinning guided the analysis of phase II (Methods section) |
| *Participant selection* | | | |
| Sampling | 10 | How were participants selected? e.g. purposive, convenience, consecutive, snowball | Purposive and convenience sampling through NGOs and municipalities (Methods section) |
| Method of approach | 11 | How were participants approached? e.g. face-to-face, telephone, mail, email | Participants were approached via researchers at seminars (face-to-face), municipal staff contacts (face-to-face and email), and invitations (mail) (Methods section) |
| Sample size | 12 | How many participants were in the study? | Phase I:<br>WS 1 A: 20 participants.<br>WS 1 B: 15 participants.<br>Phase II:<br>6 older people.<br>Phase III: |

| | | | |
|---|---|---|---|
| | | | WS 3 A: 13 stakeholders.<br>WS 3 B: 10 stakeholders.<br>WS 3 C: 9 stakeholders |
| Non-participants | 13 | How many people refused to participate or dropped out? Reasons? | No data were collected on who declined to participate. For practical reasons, not all participants were able to attend all WSs due to scheduling constraints. If someone declined to participate, they were not required to provide a reason for not attending the next WS. |
| *Setting* | | | |
| Setting of data collection | 14 | Where was the data collected? e.g. home, clinic, workplace | Phase I: WSs were conducted at the University.<br>Phase II: WSs were conducted in municipal locations, individual interviews at the University.<br>Phase III: WSs were conducted at the University.<br>(Methods section) |
| Presence of non-participants | 15 | Was anyone else present besides the participants and researchers? | Phase II and III: The WS leader (an external person) led the WSs, and a system developer participated as a participant in Phase III. A research administrator was present during WSs in phase II and also participated in the WSs in phase III. (Methods section) |
| Description of sample | 16 | What are the important characteristics of the sample? e.g. demographic data, date | The sample included key stakeholders relevant to the development of a digital intervention to reduce loneliness and social isolation among older people. In total, participants comprised social service staff |

| | | | |
|---|---|---|---|
| | | | (care/care unit managers, home-care staff, occupational therapist, technical coaches, and strategists), older people with personal experience of loneliness and/or social isolation (aged 68–91 years), IT-pedagogue, system developer, researchers, and research administrator.<br><br>Recruitment was purposive through municipal services, a caregiver center, an NGO for retired people, and through researchers' networks.<br><br>Workshops and interviews were conducted between autumn 2018 and autumn 2019 in central Sweden.<br><br>The older participants represented were of ethnically Swedish backgrounds; only one man participated. All had varying levels of digital literacy, from experienced daily computer users to those with limited skills and a preference for the telephone.<br><br>Participation was voluntary and uncompensated. (Methods section) |
| *Data collection* | | | |
| Interview guide | 17 | Were questions, prompts, guides provided by the | Phase I: Topics and questions that were discussed are presented. |

| | | | |
|---|---|---|---|
| | | authors? Was it pilot tested? | Phase II: Individual interviews and WSs followed an interview guide (Attachment A). Phase I and III were WSs with discussions around topics (Methods section) |
| Repeat interviews | 18 | Were repeat interviews carried out? If yes, how many? | Repeat WSs were conducted with same participants (Phase I and III). Individual interviews were followed up by WS 1A (Methods section) |
| Audio/visual recording | 19 | Did the research use audio or visual recording to collect the data? | Audio recordings were used for interviews and WSs phase II and III. Visual recordings were used in WSs phase II. (Methods section) |
| Field notes | 20 | Were field notes made during and/or after the interview or focus group? | Field notes were taken during WSs in all phases. Mind maps served as field notes in phase II. (Methods section) |
| Duration | 21 | What was the duration of the inter views or focus group? | Duration of interviews and WSs are presented (Methods section) |
| Data saturation | 22 | Was data saturation discussed? | Data saturation was discussed throughout the phases; similarities were found between the mind maps in phase II (Results section) |
| Transcripts returned | 23 | Were transcripts returned to participants for comment and/or correction? | Transcripts were not returned to participants. |
| **Domain 3: analysis and findings** | | | |
| *Data analysis* | | | |
| Number of data coders | 24 | How many data coders coded the data? | The first author coded the data in phase II (Methods section) |
| Description of the coding three | 25 | Did authors provide a description of the coding tree? | No. Several citations are provided for transparency (Results section for phase II). |

| | | | |
|---|---|---|---|
| Derivation of themes | 26 | Were themes identified in advance or derived from the data? | Themes were derived from the data using thematic analysis in Phase II (Results section for phase II). |
| Software | 27 | What software, if applicable, was used to manage the data? | No software for data analysis was used. |
| Participants checking | 28 | Did participants provide feedback on the findings? | Feedback was provided as part of the iterative process in phases I-III. |
| *Reporting* | | | |
| Quotations presented | 29 | Were participant quotations presented to illustrate the themes/findings? Was each quotation identified? e.g. participant number | Participant quotations, with indication of participation in interview or workshop, are presented throughout the Results section for phase II. |
| Data and findings consistent | 30 | Was there consistency between the data presented and the findings? | Findings are supported by data and quotations in phase II and similarities between themes identified in phase II and mind maps (Results section). |
| Clarity of major themes | 31 | Were major themes clearly presented in the findings? | Major themes are presented in tables and narrative for phase II (Results section). |
| Clarity of minor themes | 32 | Is there a description of diverse cases or discussion of minor themes? | All themes identified are presented at the same level (no minor themes presented). |

*Abbreviations: WS = workshops*

Developed from: Tong A, Sainsbury P, Craig J. Consolidated criteria for reporting qualitative research (COREQ): a 32-item checklist for interviews and focus groups. International Journal for Quality in Health Care. 2007. Volume 19, Number 6: pp. 349 – 357
