# Supplementary material for: Co-Designing a Digital Solution for Decreasing Loneliness and Social Isolation Among Older People in Sweden: Explorative Study
Source: JMIR Form Res. 2025 Nov 21;9:e78213. doi: 10.2196/78213 (PMC12680934; doi:10.2196/78213)
Supplement: Multimedia Appendix 2 [file formative_v9i1e78213_app2.pdf]

Multimedia Appendix 2: Interview and workshop guide phase II

Questions regarding interests

- What are your interests?
- Are there any activities that you miss doing?
- Is there anything that could help you/contribute to you performing those activities?
- Is there anything that could contribute to your social life?
- How often are you socially active/how often do you meet with other people?

Questions regarding internet use

- How often do you use the internet?
- What do you do when you are online?
- Do you meet with other people online?

- What is your overall perception of the internet? (what is good/bad?)
- What people would you like to meet online? (persons with the same interests, same age, home care staff, others)
- How would you like to communicate with other people online? (Talk, video, chat)
- What kind of activities would you want to find on the internet?
- What kind of information do you look for on the internet?
- What do you perceive as difficult on the internet?
    - Why is that?
- Do you perceive the internet to be safe? (why/why not?)
    - How could safety be improved?
- How could a web-based platform be designed to appeal to older people experiencing loneliness or who are not socially active?
