# Supplementary material for: Co-Designing a Digital Solution for Decreasing Loneliness and Social Isolation Among Older People in Sweden: Explorative Study
Source: JMIR Form Res. 2025 Nov 21;9:e78213. doi: 10.2196/78213 (PMC12680934; doi:10.2196/78213)
Supplement: Multimedia Appendix 3 [file formative_v9i1e78213_app3.pdf]

Multimedia Appendix 3: Figure S1 and S2 representing the mind maps developed during WS in phase II

Mind maps developed during WS in phase II.

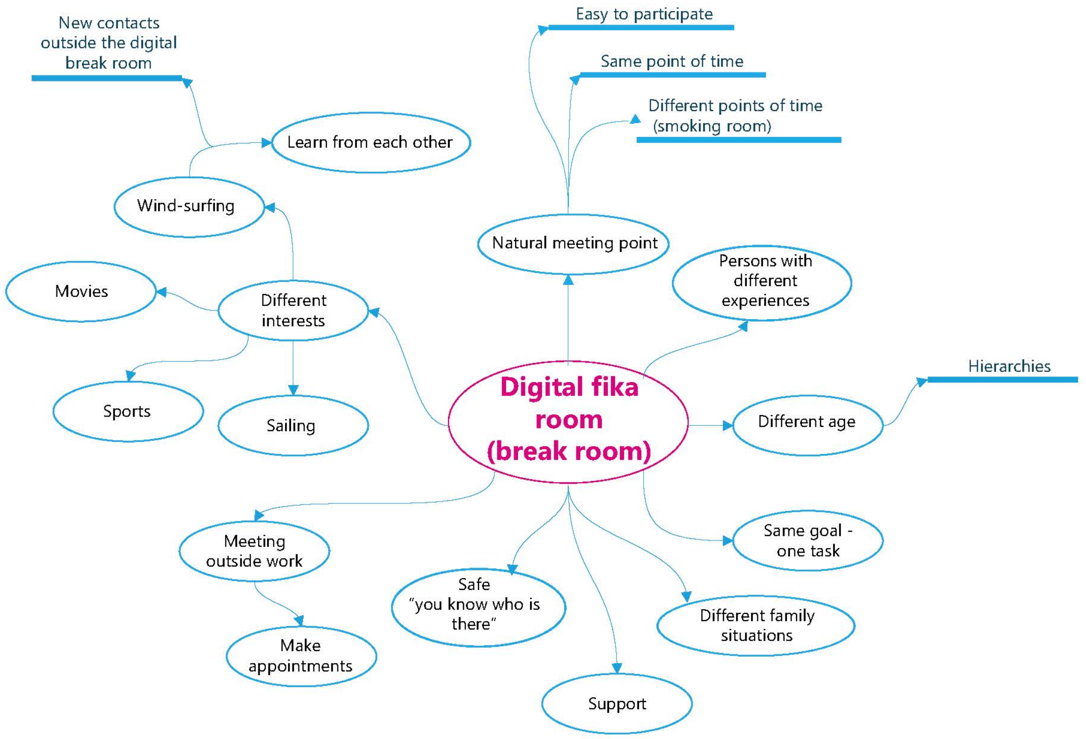

Figure S1. Mind map from WS 2 A (phase II) translated from Swedish. The workshops were conducted with older persons as part of exploring needs and preferences related to loneliness and social isolation. The mind map illustrates key discussion topics and ideas generated by participants, which informed the subsequent development of the Fik@ room.

Multimedia Appendix 3: Figure S1 and S2 representing the mind maps developed during WS in phase II

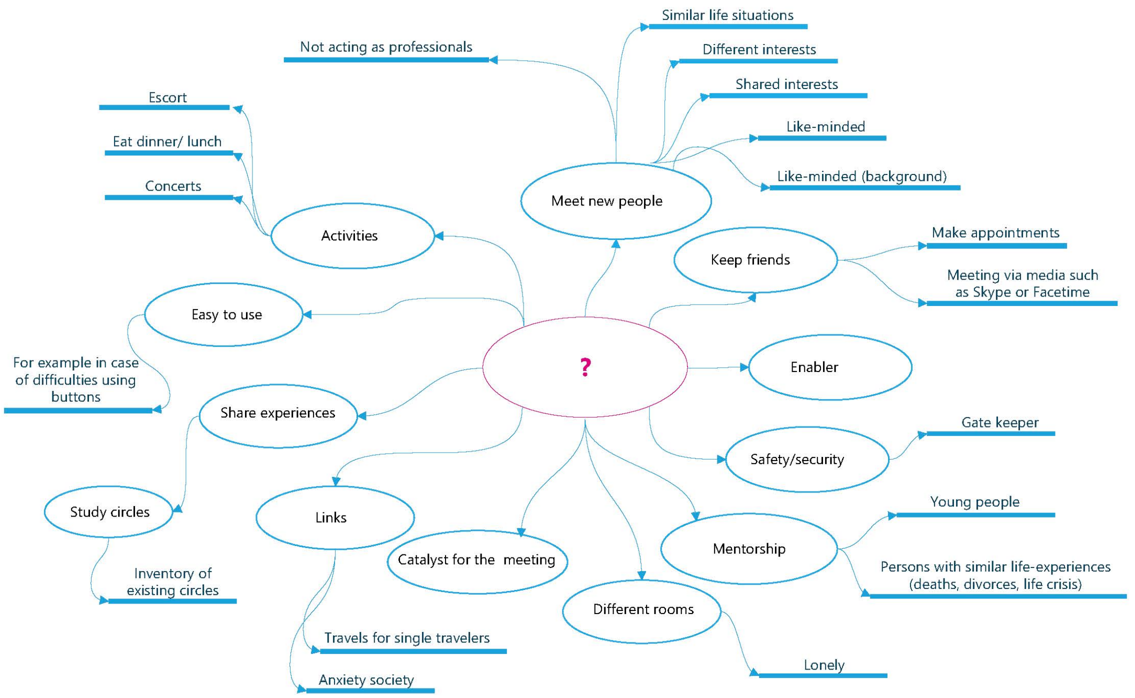

Figure S2. Mind map from WS 2 B (phase II) translated from Swedish. The workshops were conducted with older persons as part of exploring needs and preferences related to loneliness and social isolation. The mind map illustrates key discussion topics and ideas generated by participants, which informed the subsequent development of the Fik@ room.
