# Supplementary material for: Co-Designing a Digital Solution for Decreasing Loneliness and Social Isolation Among Older People in Sweden: Explorative Study
Source: JMIR Form Res. 2025 Nov 21;9:e78213. doi: 10.2196/78213 (PMC12680934; doi:10.2196/78213)
Supplement: Multimedia Appendix 4 [file formative_v9i1e78213_app4.pdf]

Multimedia Appendix 4: URL to demo of final visual interface

Link to one of the instructional videos developed to demonstrate how to navigate the Fik@ room. The video shows the final visual interface of the Fik@ room: https://play.mdu.se/media/t/0_qtvfmgib

If the link expires, please contact the corresponding author to request the demo file.
